# Dynamic Voice Optimization After Type I Thyroplasty Using a Novel Adjustable Implant: A Prospective Longitudinal Study

**DOI:** 10.3390/jcm15134927

**Published:** 2026-06-25

**Authors:** Nadhirah Mohd Shakri, Mawaddah Azman, Qi Shen Chua, Ahmed Geneid, Marina Mat Baki

**Affiliations:** 1 Department of Otorhinolaryngology-Head and Neck Surgery, Faculty of Medicine, Universiti Kebangsaan Malaysia, Bandar Tun Razak, Kuala Lumpur 56000, Malaysia; nadhirahshakri@gmail.com (N.M.S.); mawaddah@ukm.edu.my (M.A.); qishenchua99@gmail.com (Q.S.C.); 2 Hospital Canselor Tuanku Muhriz, Universiti Kebangsaan Malaysia, Bandar Tun Razak, Kuala Lumpur 56000, Malaysia; 3 Department of Otorhinolaryngology and Phoniatrics-Head and Neck Surgery, Helsinki University Hospital and University of Helsinki, Kasarmikatu 11-13, 00130 Helsinki, Finland; ahmed.geneid@hus.fi

**Keywords:** voice disorder, voice surgery, phonosurgery, laryngology, medialization thyroplasty, vocal fold paralysis, dysphonia

## Abstract

**Objective**: To evaluate the clinical outcome, safety and efficacy of the APrevent Vocal Implant System (VOIS) in patients with unilateral vocal fold paralysis (UVFP), with particular emphasis on the timing and impact of postoperative saline adjustments. **Methods**: This retrospective−prospective longitudinal study included 11 patients with chronic UVFP who underwent VOIS medialization thyroplasty (MT) under local anesthesia (*n* = 2) and general anesthesia (*n* = 9). Multidimensional voice parameters were analyzed preoperatively and at 1, 3, 6, and 12 months postoperatively. Statistical analyses included the Friedman test for repeated measures and the comparison of outcomes between pre- and each postoperative timepoints was evaluated with the Wilcoxon signed-rank test. **Results**: Significant and sustained improvements were observed across all multidimensional voice parameters. Mean mVHI-10 decreased from 31.7 ± 4.5 preoperatively to 5.8 ± 5.1 at 12 months, while mean MPT increased from 7.1 ± 3.8 to 14.4 ± 4.5 s (*p* < 0.05, r > 0.7). Acoustic parameters, including jitter, shimmer, and NHR, demonstrated progressive improvement over 12 months. A high proportion of patients (72.73%) underwent postoperative saline adjustment at a mean interval of 6.23 ± 1.23 months, beyond the early postoperative edema phase, with each adjustment yielding further enhancement in voice outcomes. No major complications, including airway obstruction or hematoma, were observed. **Conclusions**: VOIS MT is safe and effective, providing sustained improvements in multidimensional voice outcomes. The ability to perform postoperative saline adjustments enables dynamic optimization of glottal closure, reducing the need for revision surgery and addressing evolving laryngeal biomechanics. These findings support VOIS as a flexible, adjustable alternative to static medialization techniques and provide dynamic voice optimization in patients with UVFP.

## 1. Introduction

Unilateral vocal fold paralysis (UVFP) occurs due to insults or injuries to the vagus nerve, or its branch, the recurrent laryngeal nerve (RLN) [1,2]. Unfavorable position of the paralyzed vocal fold and presence of atrophy result in a phonatory gap, causing a breathy and easily fatigued voice due to glottic insufficiency [3]. Symptomatic patients with persistent UVFP of more than 6 months shall be offered permanent surgical interventions [4]. Although the optimal surgical approach to improve the glottal gap in chronic UVFP remains to be established, our local retrospective study comparing non-selective laryngeal reinnervation (NSLR) and type 1 thyroplasty with polytetrafluoroethylene (Gore-tex) implant demonstrated comparable outcomes [4]. Type 1 thyroplasty, which is also known as medialization thyroplasty (MT), involves creating a window in the thyroid cartilage through which an implant or other material is inserted to medialize the paralyzed vocal fold [5,6]. It has become a well-established phonosurgical technique for managing UVFP owing to its demonstrable and long-lasting improvement of vocal performance [7]. Over the years, a variety of materials have been used, such as Montgomery implants, Gore-tex, and titanium vocal fold medialization implant (TVFMI) [8,9,10].

However, these static implants do not allow postoperative adjustments, which can limit their effectiveness over time, especially as the paralyzed vocal fold may undergo atrophy, leading to a deterioration in voice quality. The reported revision rates for MT range from 4.5% to 16%, with the most common revision involving replacement with a larger implant [11]. Our local study demonstrated a revision rate of 5.4% following Gore-Tex MT, with indications including gradual dysphonia starting five months postoperatively and worsening aspiration occurring up to three years after the initial surgery [12]. This suggests that under-correction is common and highlights the challenges surgeons face due to perioperative vocal fold edema, which may limit accurate assessment of medialization during the initial procedure. Furthermore, MT may not adequately address the posterior glottal gap, often necessitating an additional arytenoid adduction procedure. This procedure requires deep dissection behind the thyroid cartilage and carries risks of laryngeal mucosal injury as well as airway compromise due to hemilaryngeal edema or hematoma. Nito et al. reported postoperative airway compromise in 27% of patients undergoing arytenoid adduction, with 1.6% requiring emergency tracheotomy [13]. Variability in thyroid cartilage anatomy may also affect the accuracy of fenestration and implant positioning. In females, a wider thyroid cartilage angle results in a more laterally positioned posterior segment, which may limit effective medialization [14]. This anatomical difference has been associated with less favorable outcomes following Montgomery MT, with significantly less improvements in maximum phonation time (MPT) observed in female patients compared to males [15]. In addition, studies have demonstrated variability between the intended and actual fenestration site, with optimal voice outcomes linked to a more infero-anterior window position, which was predominantly achieved in males and more difficult to achieve in females [16]. Collectively, these findings highlight the anatomical and technical limitations of the Montgomery system in female patients, leading to less predictable outcomes and underscoring the need for more adaptable medialization implants.

The APrevent^®^ Vocal Implant System (VOIS) was developed to address these limitations. Its design incorporates an inflatable silicone cushion that medializes the arytenoid toward the midline when inflated, reducing the need for additional arytenoid adduction procedures. It offers a distinct advantage over static implants by permitting postoperative re-adjustment of the filling volumes [17]. In addition, VOIS is designed to be patient-specific, available in multiple sizes, which is based on individual anatomical characteristics rather than sex, further supporting its suitability across a diverse patient population [18]. Early studies have demonstrated promising short-term outcomes with VOIS [17,18] and subsequent reports with follow-up beyond one year have shown sustained voice improvement [19]. In the existing literature, postoperative adjustments are mostly performed at approximately seven weeks following surgery, coinciding with the resolution of laryngeal edema [18,19]. However, these studies primarily focus on early postoperative optimization, while the frequency, timing, and clinical significance of later adjustments remain poorly understood. It is unclear whether voice deterioration occurs beyond the early postoperative period and how delayed adjustments contribute to long-term voice optimization.

First in Southeast Asia, this prospective longitudinal study evaluates multidimensional voice outcomes following VOIS MT, focusing on postoperative adjustment patterns and late voice changes in patients with UVFP, to highlight the clinical value of this dynamic, adjustable medialization approach for long-term management.

## 2. Materials and Methods

### 2.1. Study Design

This is a retrospective−prospective longitudinal study of patients with chronic UVFP who underwent MT with the novel APrevent^®^ VOIS (APrevent Medical Ltd., Taiwan, China) at a tertiary teaching hospital. Multidimensional voice assessments were performed preoperatively and postoperatively at 1-, 3-, 6- and 12 months. One patient who underwent surgery in January 2023 as part of routine clinical care was included retrospectively and subsequently completed follow-up assessments according to the study protocol during the approved study period, from January 2023 until January 2024. The remaining patients were prospectively recruited using convenience sampling from September 2023 until February 2025 and underwent the same assessment schedule. The exclusion criteria were a history of cordectomy and the presence of structural vocal fold lesions or high vagal paralysis with poor laryngeal sensation. The study was conducted in accordance with the Declaration of Helsinki and approved by the Universiti Kebangsaan Malaysia Ethics Committee (Approval No. JEP-2023-674; Study Code No FF-2023-410; Date of Approval: 26 September 2023). Written informed consent was obtained from all participants prior to study participation.

### 2.2. Study Instruments

#### APrevent^®^ Vocal Implant System (VOIS)

VOIS, which is available in four sizes, consists of three components: titanium housing with integrated port-chamber and port-membrane, fixation plate with screw, and an in- and deflatable silicone cushion. The VOIS was inserted through a window on the ipsilateral thyroid cartilage ala by performing MT. In cases performed under general anesthesia (GA), a laryngeal mask airway (LMA) was used. Following insertion of VOIS, transnasal flexible videolaryngoscopy was performed via the LMA to assess glottal closure, arytenoid position and airway patency. Medialization was achieved by injecting 0.9% saline into the port-chamber using a 1 mL syringe with a 24-gauge needle to inflate the silicone cushion until medialization of the paralyzed vocal fold and improvement in arytenoid position were achieved (Video 1). In cases performed under local anesthesia (LA), the adjustment procedure involved real-time phonation. Patients were instructed to phonate the vowel “ee”, while incremental injections or aspirations of normal saline were made to the silicone cushion in 0.02 mL steps.

### 2.3. Multidimensional Voice Parameters

#### 2.3.1. Subjective Evaluation

Patients’ self-perceived voice outcomes were assessed using the Bahasa Malaysia Voice Handicap Index-10 (mVHI-10) questionnaire [20]. The questionnaire was completed by the patients to reflect the extent to which their quality of life was impacted by the voice disorder. A mVHI-10 score of more than 7.5 is considered abnormal for our population, as described by Idris et al. [21]. In addition, perceptual voice evaluation by a laryngologist using a scale comprising grade of hoarseness, roughness, breathiness, asthenia, and strain (GRBAS) scale [22]. In the current study, the breathiness component is documented as proposed by Mattei et al. [23]. Laryngostroboscopy was performed as routine assessment during follow-up visits at each timepoint, to assess the vocal fold and arytenoid positions, phonatory gap and rule out any structural disease.

#### 2.3.2. Objective Evaluation

Acoustic analysis is performed by using a non-invasive computer software OperaVOX^TM^ Multi (On Person RApid VOice eXaminer, Oxford Research Wave Ltd., Oxford, UK), portable voice analysis software running on iPod touch 6th generation (Apple, Cupertino, CA, USA) to measure the fundamental frequency (F0), jitter % (cycle-to-cycle variation in pitch), shimmer % (cycle- to-cycle variation in amplitude), and noise-to-harmonic ratio (NHR) to measure the quality of recorded voices [24]. MPT was used to assess the glottic efficiency by measuring the longest duration of the patients’ ability to sustain a phonation /a/. Longer MPT indicates more stable and better voice endurance.

### 2.4. Statistical Analysis

Demographic data were analyzed and reported descriptively. Data analysis was performed using SPSS version 29.0.1.0. Comparisons of multidimensional voice parameters across multiple timepoints (preoperative and postoperative at 1, 3, 6, and 12 months) were performed using the Friedman test. Kendall’s W was calculated as the effect size to quantify the magnitude of change across repeated measures, with 0.1, 0.3, and 0.5 representing small, moderate, and large effects, respectively. Pairwise comparisons between preoperative and each postoperative timepoint for all voice parameters were analyzed using the Wilcoxon signed-rank test. A *p*-value of <0.05 was considered statistically significant, with Bonferroni correction applied for multiple comparisons (adjusted $\propto \;=\frac{0.05}{3}=0.017$). The effect size r was computed (r = Z/√N) to evaluate the magnitude of postoperative change, where effect sizes of 0.1, 0.3, and 0.5 are interpreted as small, medium, and large, respectively.

## 3. Results

### 3.1. Demographic Data

The patients’ demographic characteristics are summarized in Table 1. A total of 11 patients with UVFP underwent VOIS MT, including two under LA and nine under GA. The cohort comprised seven females and four males, with a median age of 40 years. Eight patients had left-sided UVFP and three had right-sided UVFP. The mean (SD) duration of palsy was 54.18 (118.15) months, with median being 20 months. Notably, one patient had a longstanding history of left-sided UVFP for 34 years, attributed to cardiothoracic surgery during childhood. The most common etiology was thyroid surgery (*n* = 6), followed by idiopathic (*n* = 2), with one case each related to tuberculosis (*n* = 1), excision of vagal paraganglioma (*n* = 1) and cardiothoracic surgery (*n* = 1). Three male patients received VOIS-M (medium size) and one male received VOIS-S (small size), while two VOIS-S and five VOIS-XS (x-small size) devices were implanted in the six female patients. All patients received intraoperative saline injection.

### 3.2. Multidimensional Voice Parameters

The multidimensional voice outcomes are summarized in Table 2. The mean (SD) mVHI-10 scores improved significantly across timepoints, decreasing from a baseline mean of 31.73 (4.54) to 12.88 (10.09), 12.50 (9.34), 9.36 (7.29), and 5.77 (5.09) at the 1-, 3-, 6-, and 12-month follow-ups, respectively (*p* < 0.01). The proportion of patients with a breathiness score of ≥2 on the GRBAS scale was 66.7% at baseline, which decreased to 11.1% at 1 month and further reduced to 0% at the 3-, 6-, and 12-month postoperative follow-ups. The mean (SD) of MPT also improved significantly across timepoints, increasing from a baseline value of 7.09 (3.83) seconds to 11.67 (6.01), 12.71 (2.31), 13.21 (4.17), and 14.40 (4.50) seconds at the 1-, 3-, 6-, and 12-month intervals, respectively (*p* < 0.001). The mean (SD) baseline values for jitter, shimmer, and noise-to-harmonic ratio (NHR) were 5.53 (3.32), 10.76 (7.19), and 0.63 (0.77), respectively. At the 1-, 3-, 6-, and 12-month follow-ups, the jitter values were 2.85 (2.40), 2.71 (2.31), 2.36 (1.73), and 2.22 (2.15), respectively. The corresponding shimmer values were 14.10 (21.52), 5.81 (2.46), 5.75 (2.40), and 4.97 (2.75), while the NHR values were 0.15 (0.28), 0.17 (0.24), 0.13 (0.23), and 0.07 (0.07), respectively. Although improvements were observed in the acoustic parameters, only jitter showed statistically significant improvements across timepoints (Table 2).

### 3.3. Comparison of Baseline and Postoperative Voice Parameters

**Figure 1:**
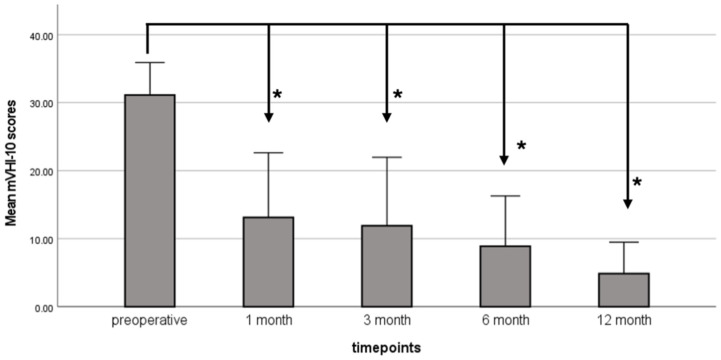
Comparison of Bahasa Malaysia voice handicap index-10 (mVHI-10). Wilcoxon signed-rank tests demonstrated statistically significant improvement at all postoperative timepoints compared with baseline (* *p* < 0.05).

**Figure 2:**
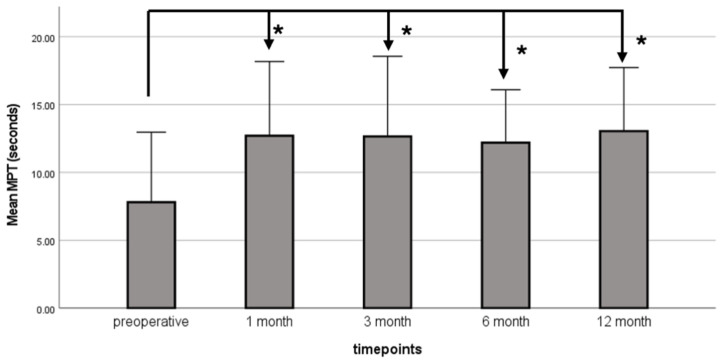
Comparison of maximum phonation time (MPT). Wilcoxon signed-rank tests comparing preoperative and postoperative MPT demonstrated statistically significant improvement at all timepoints after VOIS medialization thyroplasty (* *p* < 0.05).

**Figure 3:**
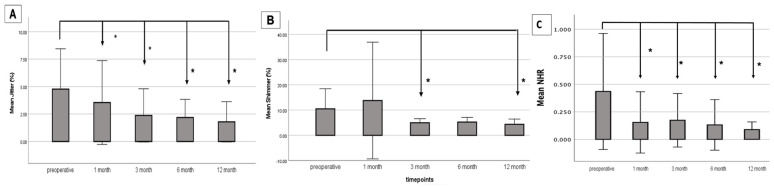
Comparison of acoustic parameters. Wilcoxon signed-rank test for acoustic parameters from preoperative to multiple timepoints post medialization thyroplasty with VOIS. (**A**) Jitter. (**B**) Shimmer. (**C**) Noise-to-harmonic ratio (NHR). Jitter and NHR showed statistically significant improvement from preoperative to all timepoints (* *p* < 0.05). Shimmer showed statistically significant improvement at postoperative 3- and 12-month.

The multidimensional voice parameters were compared between baseline and each postoperative timepoints. Wilcoxon signed-rank tests demonstrated statistically significant improvements in mVHI-10 from baseline to all postoperative timepoints (*p* < 0.05, r > 0.7) (Figure 1). Similarly, MPT showed significant improvements from baseline to all postoperative timepoints (*p* < 0.05, r > 0.7) (Figure 2). Jitter improved significantly from baseline to all timepoints (*p* < 0.05, r > 0.6) (Figure 3A). Shimmer demonstrated significant improvement at 3-month (*p* = 0.01, r = 0.75) and 12-month (*p* = 0.01, r = 0.77), but not at 1-month (*p* = 0.89, r = 0.14) and 6-month (*p* = 0.07, r = 0.54) (Figure 3B). NHR demonstrated significant improvement from baseline to all timepoints (*p* < 0.05, r > 0.6) (Figure 3C).

### 3.4. Postoperative Saline Adjustment

Eight patients (72.73%) required postoperative saline adjustments. The indications for adjustment included deterioration of voice and the presence of a glottal gap on laryngostroboscopy. The mean (SD) time to adjustment was 6.33 (2.65) months, ranging from 4 to 12 months after surgery, with a median of 6 months (Table 3). Most patients underwent transcutaneous saline adjustment with or without ultrasound guidance. However, one patient required small neck incision and exploration at 4 months, following persistent minimal improvement in voice parameters. Multiple attempts at saline adjustment were initially made under LA, but the external membrane port could not be identified. Ultrasound-guided adjustments were also conducted; however, despite successful localization of the membrane port, needle penetration was unsuccessful. Exploration revealed inadequate snapping of the implant’s anterior flange into the cartilage, likely from the initial procedure. The same implant was then repositioned, after which the patient experienced an uneventful recovery. No implant migration was observed in our cohort. Post-adjustment laryngostroboscopy demonstrated improved glottal closure in all patients. All patients underwent a single postoperative adjustment except for one patient, who required two adjustments at 4 and 6 months postoperatively. Interestingly, all cases demonstrated discrepancies between the volume of saline aspirated and the initial intraoperative injection volume.

## 4. Discussion

MT remains a well-established surgical option for UVFP; however, achieving sustainable voice improvements while accommodating dynamic anatomical and biomechanical changes over time remains a challenge. This prospective longitudinal study evaluated multidimensional voice parameters and postoperative adjustability of VOIS over a 12-month follow-up period. Our cohort demonstrated significant and sustained improvements across multidimensional voice outcomes, with gains evident as early as 1 month postoperatively and remaining stable throughout the 12-month follow-up period. The progressive improvement in mVHI-10 scores across postoperative timepoints reflects a substantial gain in patients’ perceived voice quality. Although a VHI-10 score below 11 is generally considered within the normal range [25], the established cut-off point for mVHI-10 in our population is 8 [21]. In the present cohort, the mean mVHI-10 score achieved a near-normal range by 6 months postoperatively and normalized by 12 months, suggesting sustained improvement in patient-reported voice outcomes over time. When compared with our previous cohort undergoing Gore-Tex MT, the current cohort demonstrated lower mean mVHI-10 scores at similar follow-up intervals, despite comparable baseline scores [4]. Although no formal statistical comparison was performed, these findings suggest greater patient-perceived voice improvement following VOIS MT, likely related to its postoperative adjustability. The normalization of mVHI-10 by 12 months reflects the effect of saline adjustments, supporting their role in progressive voice optimization.

The lack of statistically significant improvement in shimmer at 1 and 6 months may be explained by different factors. At 1 month, postoperative edema and ongoing tissue healing may contribute to instability in vocal fold vibration, thereby affecting amplitude-based acoustic measures such as shimmer despite adequate glottic closure. At 6 months, the mean timing of postoperative VOIS adjustment in our cohort may have introduced a transient change in glottic closure and vibratory characteristics, potentially masking the overall improvement trend. As shimmer reflects amplitude stability, these dynamic postoperative changes may have influenced the observed outcomes at these timepoints. In contrast, jitter demonstrated significant improvement throughout follow-up, indicating sustained enhancement in cycle-to-cycle frequency stability following medialization. Similarly, patient-reported voice outcomes, as measured by the mVHI-10, showed progressive and statistically significant improvement at all postoperative assessments. Although NHR demonstrated marked improvement compared with baseline, statistical significance was not maintained at 12 months. This may be related to the small cohort size and inherent variability of acoustic measurements, which could have limited the power to detect differences at individual timepoints. Discrepancies between objective acoustic measures and subjective voice outcomes are well recognized in the literature, as each modality captures distinct aspects of voice function and patient experience [26]. These findings underscore the importance of a multidimensional approach when evaluating voice outcomes following medialization thyroplasty.

Among the eleven patients in our cohort, seven achieved an MPT exceeding 10 s at 6 months postoperatively, increasing to nine patients at 12 months. Notably, eight patients surpassed the sex-specific normative MPT values for our population, which are 21.41 (±6.85) seconds for males and 18.05 (±5.06) seconds for females [27]. Overall, our study demonstrated longer mean MPT values compared with other studies evaluating VOIS MT [19]. The relatively better MPT outcomes observed in our cohort may be attributed to the younger median age of our patients (40 years), compared with the cohorts in those studies, where the mean age exceeded 60 years [18,19]. Younger patients generally exhibit better vital lung capacity and ability to generate adequate subglottic pressure, both of which contribute to more sustained phonation and longer MPT [28].

Previous studies have reported sex-related differences in outcomes following MT, with female patients demonstrating significantly lesser improvements in MPT compared to males [15]. These differences have been attributed to anatomical variations in thyroid cartilage structure, including increased lamina angulation and a more laterally positioned posterior segment, which may limit effective medialization. Although the female thyroid cartilage is generally smaller, the greater lateral displacement of the posterior framework may require deeper or larger prostheses to achieve adequate glottic closure. In the Montgomery Implant System Thyroplasty, implant design is sex-specific, in which the male-designated implants are bigger and provide greater endolaryngeal depth than female implants of the same nominal sizes [29]. Consistent with this, Oishi reported that female patients undergoing revision thyroplasty experienced improved vocal outcomes after conversion to male-designated implants, likely due to enhanced posterior glottic medialization [30]. VOIS adopts a measurement-based intraoperative sizing strategy rather than relying on sex-based presets. Implant size is determined according to the measured distance from the anterior midline to the posterior thyroid cartilage. Previous VOIS studies have demonstrated a general trend toward larger implant sizes (M, L) in male patients and smaller sizes (XS, S) in female patients [18,19]. In our cohort, most implant selections followed similar distribution, except for one male patient who required a VOIS-S implant. This finding highlights that implant requirements are not solely determined by sex but instead reflect individual anatomical variability. Collectively, these observations underscore the limitations of sex-based sizing strategies and support a patient-specific, measurement-guided approach, as employed in VOIS to allow precise medialization.

Table 4 demonstrates a comparison of patient characteristics and multidimensional voice parameters with other VOIS studies. However, there were notable differences in baseline characteristics across studies, including patient age, anesthetic approach, and follow-up duration, with 12 months in our study compared with shorter-term follow-up or up to 26 months in other reports. These variables may influence postoperative outcomes and adjustment rates, and therefore, the comparisons should be interpreted as contextual rather than directly equivalent between cohorts. Most procedures in our cohort were performed under GA. Although LA allows intraoperative voice fine-tuning, the VOIS system provides postoperative adjustability, enabling optimization of medialization after surgery. The ability to perform VOIS MT under GA is particularly advantageous in patients with anxiety or fear of awake procedures, which is frequently encountered in our outpatient setting. GA is also preferred when NSLR is initially planned; however, in cases where reinnervation is not feasible, such as in the absence of a donor nerve, conversion to VOIS thyroplasty remains a viable option.

Our cohort demonstrated the highest reported rate of postoperative saline adjustment (72.73%) following VOIS thyroplasty, compared with 38.5% and 28% reported in previous VOIS series [18,19]. Furthermore, while adjustments in prior studies were typically performed approximately 7 weeks postoperatively and were largely attributed to voice deterioration following resolution of early postoperative edema, the median time to adjustment in our cohort was 6 months (Table 4). This delayed timing suggests that the need for adjustment was not primarily driven by transient postoperative changes. Rather, UVFP is a dynamic condition characterized by progressive neuromuscular and biomechanical alterations, including ongoing thyroarytenoid muscle atrophy, which may result in late deterioration of glottic competence and voice quality beyond the early postoperative period. The higher adjustment rate observed in our series is likely multifactorial. First, the majority of procedures were performed under GA, where intraoperative phonatory feedback was unavailable; consequently, medialization was guided primarily by endoscopic assessment, which may contribute to subtle under-correction in selected cases. As this represents our initial institutional experience with VOIS implantation, a learning curve effect, including progressive refinement in implant positioning and saline adjustment strategies, may also have influenced the need for subsequent optimization. Second, the relatively younger age and higher vocal demand of patients in our cohort may have increased sensitivity to subtle changes in voice performance, resulting in earlier recognition of functional deterioration. Finally, the availability of an adjustment mechanism, together with a proactive follow-up protocol in our tertiary voice center, likely facilitated a more responsive approach to postoperative optimization. Unlike static implants, where comparable changes may not justify revision surgery, the ease and safety of VOIS adjustment allow subtle voice deterioration to be addressed as part of longitudinal voice management rather than in response to implant failure. Collectively, these findings suggest that the delayed timing and higher frequency of postoperative adjustments reflect the dynamic nature of UVFP, procedural and learning-curve factors, and an active optimization strategy enabled by an adjustable implant system. Importantly, the observed adjustment rate should be interpreted as a feature of longitudinal voice optimization rather than a failure. Due to the small cohort size, subgroup analyses comparing patients requiring postoperative adjustment versus those who did not were not performed, as such analyses would be underpowered.

Most saline adjustments were performed under ultrasound guidance to confirm the needle entry angle after palpation of the port and to facilitate precise saline adjustment. In several cases, the aspirated saline volume appeared smaller than the amount injected intraoperatively (Table 3). This discrepancy may reflect a combination of technical and measurement factors, including minimal injection volumes, syringe–needle dead space, and potential air within the syringe during injection or aspiration. Similar minor volume variations have been described in other inflatable saline implants [31,32]. Given that VOIS inflation volumes are typically <0.3 mL, even small technical variations may result in apparent differences in aspirated volume. Further studies with larger cohorts and longer follow-up are needed to identify predictors of postoperative adjustment requirements and to better characterize long-term implant volume dynamics and saline resorption rate.

This study has several limitations, including its single-center design and single-arm design without a control group. Additionally, the absence of randomization introduces the potential for selection and outcome assessment bias. Furthermore, the relatively small sample size (*n* = 11) in our cohort may limit the statistical power. Future studies should include larger multi-center cohorts and comparative designs to evaluate the effectiveness of VOIS against other implant materials. Comparative analyses involving NSLR or additional procedures such as arytenoid adduction would also provide valuable insights into optimal treatment strategies for UVFP.

## 5. Conclusions

VOIS medialization thyroplasty performed under either GA or LA is safe and yields favorable multidimensional voice outcomes in patients with UVFP. Postoperative percutaneous saline adjustment allows further optimization of glottal configuration without the need for implant replacement. These findings highlight the clinical value of a dynamic, adjustable implant system in accommodating evolving laryngeal biomechanics compared with static medialization techniques. While this approach may potentially reduce the need for revision procedures, this requires confirmation in future comparative studies.

## Figures and Tables

**Table 1:** Demographic data of patients who underwent VOIS medialization thyroplasty (*n* = 11).

**Age, Year**		
Mean (SD)	43.64 (12.40)	
Median (Min, Max)	40 (29,64)	
**Gender**	Male	Female
	4	7
**Side of UVFP**	Right	Left
	3	8
**Duration of palsy, month**		
Mean (SD)	54.18 (118.16)	
Median (min, max)	20 (4408)	
**Etiologies**	Thyroid surgery (*n* = 6)	
	Idiopathic (*n* = 2)	
	Cardiothoracic surgery (*n* = 1)	
	Tuberculosis (*n* = 1)	
	Excision of vagal paraganglioma surgery (*n* = 1)	

VOIS: APrevent^®^ Vocal Implant System, SD: standard deviation, min: minimum, max: maximum, UVFP: unilateral vocal fold paralysis.

**Table 2:** Multidimensional voice parameters before and after medialization thyroplasty with VOIS (*n* = 11).

Outcome Measure	Preoperative Mean (SD)	1 Month Mean (SD)	3 Months Mean (SD)	6 Months Mean (SD)	12 Months Mean (SD)	*p*-Value (Kendall’s W)
mVHI-10	31.73 (4.54)	12.88 (10.09)	12.50 (9.34)	9.36 (7.29)	5.77 (5.09)	<0.01 * (0.59)
MPT (seconds)	7.09 (3.83)	11.67 (6.01)	12.71 (5.29)	13.21 (4.17)	14.40 (4.50)	<0.001 * (0.71)
Jitter (%)	5.53 (3.32)	2.85 (2.40)	2.71(2.31)	2.36 (1.73)	2.22 (2.15)	0.04 (0.35) *
Shimmer (%)	10.76 (7.19)	14.10 (21.52)	5.81 (2.46)	5.75 (2.40)	4.97 (2.75)	0.17 (0.10)
NHR	0.56 (0.57)	0.15 (0.28)	0.20 (0.27)	0.15(0.27)	0.07 (0.07)	0.41 (0.31)

*p*-values compare the multidimensional voice parameters across multiple timepoints using the Friedman test (* significant *p* < 0.05). Kendall’s W values represent the effect size, indicating the standardized difference across all pre- and postoperative timepoints. VOIS: APrevent^®^ Vocal Implant System, SD: standard deviation, mVHI-10: Bahasa Malaysia voice handicap index-10, MPT: maximum phonation time, NHR: noise-to-harmonic ratio.

**Table 3:** Profile of patients requiring postoperative saline adjustment.

Patient	Duration from Surgery (Month)	Intraoperative Saline Amount (mL)	Amount Aspirated (mL)	Amount Infiltrated (mL)	Approach
A	8	0.10	0.05	0.15	Transcutaneous
B	6	0.14	0.07	0.12	Transcutaneous
C	4	0.11	0.10	0.15	Transcutaneous USG-guided
D	12	0.20	0.05	0.15	Transcutaneous USG-guided
E	4	0.10	0.05	0.07	Neck exploration
F	8	0.10	0.07	0.10	Transcutaneous USG-guided
G	5	0.10	0.02	0.14	Transcutaneous USG-guided
H	4	0.15	nil	0.15	Transcutaneous USG-guided
H	6	0.15	0.05	0.17	Transcutaneous

USG: ultrasound, mL: milliliter.

**Table 4:** Comparison of patient characteristics and multidimensional voice parameters in VOIS studies.

Study		Hsieh et al. [14] (2021)	Huang et al. [15] (2023)	Current Study (2025)
Number of patients		13 (8 males, 5 females)	14 (9 males, 5 females)	11 (4 males, 7 females)
Mean age (year) ± SD		62 (42–75)	62.4 ± 10.8	43.64 ± 12.40
Mean duration of UVFP (month)		-	-	54.18 ± 118.16
Duration of follow up (month)		6	26 ± 8.0	12
Anesthesia		LA	LA	9 GA, 2 LA
VOIS size		Male: M (5), L (3) Female: S (2), XS (3)	Male: M (7), L (2) Female: S (2), XS (3)	Male: M (3), S (1) Female: S (2), XS (5)
Mean MPT (seconds)	Preoperative	2.3 ± 1.6	3.3 ± 2.6	7.09 ± 3.83
	6 months	6.5 ± 4.4	7.5 ± 4.4	13.21 ± 4.17
	12 months	-	7.8 ± 3.7	14.40 ± 4.50
Mean jitter (%)	Preoperative	4.1 ± 3.0	2.91 ± 2.62	5.53 ± 3.32
	6 months	0.7 ± 0.4	0.93 ± 0.48	2.36 ± 1.73
	12 months	-	0.81 ± 0.48	2.22 ± 2.15
Mean shimmer (%)	Preoperative	1.4 ± 0.3	1.01 ± 0.53	10.76 ± 7.19
	6 months	0.5 ± 0.2	0.76 ± 0.31	5.75 ± 2.40
	12 months	-	0.66 ± 0.34	4.97 ± 2.75
Number of patients requiring postoperative adjustment (%)		5 (38.5%)	4 (28%)	8 (72.73%)
Mean duration before postoperative adjustment (SD)		7 weeks	7 weeks	6.33 ± 2.65 months

## Data Availability

The data presented in this study are available from the corresponding author upon reasonable request. The data are not publicly available due to institutional and patient confidentiality restrictions.
